# Correction: Exploring the impact of trait number and type on functional diversity metrics in real-world ecosystems

**DOI:** 10.1371/journal.pone.0317138

**Published:** 2025-01-02

**Authors:** Timothy Ohlert, Kaitlin Kimmel, Meghan Avolio, Cynthia Chang, Elisabeth Forrestel, Benjamin Gerstner, Sarah E. Hobbie, Kimberly Komatsu, Peter Reich, Kenneth Whitney

The eight author’s name is spelled incorrectly. The correct name is: Kimberly Komatsu.

The eighth author is listed out of order. The correct order is:

Timothy Ohlert^1^*, Kaitlin Kimmel^2,3^, Meghan Avolio^3^, Cynthia Chang^4^, Elisabeth Forrestel^5^, Benjamin Gerstner^1^, Sarah E. Hobbie^6^, Peter Reich^8,9,10^, Kenneth Whitney^1^, Kimberly Komatsu^7^

**1** Department of Biology, University of New Mexico, Albuquerque, NM, United States of America, **2** Mad Agriculture, Boulder, Colorado, **3** Department of Earth & Planetary Sciences, Johns Hopkins University, Baltimore, MD, United States of America, **4** Division of Biological Sciences, University of Washington, Bothell, WA, United States of America, **5** Department of Viticulture and Enology, University of California, Davis, CA, United States of America, **6** Ecology, Evolution and Behavior Department, University of Minnesota, St. Paul, MN, United States of America, **7** Smithsonian Environmental Research Center, Edgewater, MD, United States of America, **8** Department of Forest Resources, University of Minnesota, Minneapolis, MN, United States of America, **9** Institute for Global Change Biology and School for Environment and Sustainability, University of Michigan, Ann Arbor, MI, United States of America, **10** Hawkesbury Institute for the Environment, Western Sydney University, Penrith South, NSW, Australia
