# Supplementary material for: Exploring the impact of trait number and type on functional diversity metrics in real-world ecosystems
Source: PLoS One. 2022 Aug 25;17(8):e0272791. doi: 10.1371/journal.pone.0272791 (PMC9409596; doi:10.1371/journal.pone.0272791)
Supplement: S1 File — (DOCX) [file pone.0272791.s001.docx]

**Data collection protocols**

**Cedar Creek**

All trait data was collected in the ambient monoculture plots of the BioCON experiment (n = 2 plots per species for 16 species). Trait data was averaged over all measurements for the given trait if it was collected in multiple years, so our data represent long-term averages and should not be influenced by year-to-year fluctuations. Detailed protocols can be found at <https://www.cedarcreek.umn.edu/research/data/methods?e141>. Below we describe them briefly.

Our measurements for I* (the amount of light at the soil surface in monoculture) are from the Cedar Creek dataset “lpe141”. I* measurements are available for 1998 - 2019. Light meters were used to take measurements below the canopy, but above any litter from the precious year. Three measurements in each plot were taken and the average is reported in the raw data. The proportion of light reaching the meter is what was recorded such that species with higher I* values allow more light through to the soil surface.

Our measurements for R* (the amount of nitrogen in monoculture) are from the Cedar Creek dataset “nohe141”. Measurements of soil nitrate and ammonium were taken between 1998 and 2002. Soil cores (0.75 inch diameter) were taken from each plot after biomass harvest in 20 cm increments. Nitrate and ammonium were extracted from the cores using KCl. For R* we use the nitrate values from 0-20 cm depth. Species with a lower R* are able to draw down nitrate more in monoculture.

Our measurements for total root biomass are from the Cedar Creek dataset “roote141”. Data were collected from 1998 to 2020. Root cores are taken at 20 cm intervals following biomass harvest. Roots are washed and sorted into coarse, crown, and fine roots. These were then dried and weighed. For our analyses, we combine all root sections to get total root biomass.

Our measurements for root %C and %N are from Cedar Creek dataset “nre141”. Data are available from 1998 - 2018. Root cores are taken at 20 cm intervals in each plot following aboveground biomass harvest. Washed root samples were then dried and ground before being run for %C and %N. Here we use data from 0-20 cm cores where the majority of root biomass is.

Our measurements from shoot %N and shoot %C are from Cedar Creek dataset “nbe141” and are available from 1998 - 2018. Biomass is harvested from a 1m x 0.1 m strip, dried, ground and homogenized before sending out for %C and %N analyses.

Seed mass measurements are from Cedar Creek dataset “aafe120” and were taken in 2008. These measurements are from an adjacent experiment at Cedar Creek. Seed heads were collected from ten plants for each species. After drying, 10 seeds were weighed. Some species seed mass was taken from online databases, but is not clarified in the dataset which these are.

Specific leaf area measurements are from Cedar Creek dataset “phoe141” and were taken in 1998-2018. Leaves were sampled after they were measured for photosynthesis and measured using a digital image analysis program.

**Konza**

Trait data were collected in watersheds that manipulate grazing and fire frequency. We will use trait data from plots that are burned annually with no grazers that were collected between 2010 and 2012. Trait data are averaged over all measurements of a given species. Methods can be found in [1] and [2] and are outlined below.

At least 10 individuals per species were measured for each trait. Maximum plant height was measured and four leaves per individual were collected.

Two leaves of each individual were rehydrated for 24 to 48 hours after collection to obtain leaf area and wet mass. These leaves were then dried for 48 hours at 60C to obtain dry weight to calculate specific leaf area (leaf area/dry mass) and leaf dry matter content (dry mass/wet mass).

Dried leaves were ground for leaf %N and leaf %C analysis. Samples were analyzed using a Costech ESC 4010 Elemental Combustion System (Costech Analytical Technologies, Valencia, CA, USA) at Yale University’s Earth System Center for Stable Isotopic Studies.

The two leaves that were not dried were preserved in 70% ethanol solution. Dental putty was used to make impressions of the abaxial surface of two leaves from five individuals per species (N = 10 per species). Nail polish peels were used to measure the length of 6 stomata and count stomata for density. Stomatal pore index was calculated as the stomatal density multiplied by the square of the mean guard cell length.

**Sevilleta**

Trait measurements at Sevilleta closely followed the plant trait handbook of Pérez-Hariguideguy 2013. Plants were collected from across the Blue, Black, Creosote and PJ core sites at the Sevilleta. Samples were collected in 2017 summer and fall with some collections in 2018 spring for spring annuals.

Our measurements for maximum plant height were taken in 2017-2020. They are the average height (of green material) for 5 individuals per species, measured in the field.

Samples for leaf, stem, root, and isotope were haphazardly collected for 1-10 individuals per species from different locations across the Sevilleta site, primarily focused around the blue grama, black grama, creosote and pinon-juniper core sites. These samples were transported to the lab in plastic bags with moist paper towels, whereafter samples were prepared and the following measurements were taken.

For leaf trait measurements we removed 5 leaves or leaflets per plant (excising petioles), imaged them on a flatbed scanner. Leaves were then patted dry and weighed (wet mass), they were then dried at 60C to a constant weight and massed again (dry mass). Leaf dry matter content was calculated as dry mass divided by wet mass.

For specific leaf area, we used ImageJ to automatically measure the area of the scanned leaf images. Leaf area was calculated as the arithmetic average of the area of all leaves/leaflets imaged.

For stem and root dry matter content we excised a 1-3cm section of the tissue directly above (stem) and below (root) the soil interface. Sections were patted dry and weighed (wet mass), then dried at 60C to a constant weight and massed again (dry mass). Dry matter content was calculated as dry mass divided by wet mass.

Our measurements for d15N, d13C, %N, %C were taken in 2017-2020. We used foliage from up to 3 individuals per species, foliar tissues were dried to a constant weight, powered with beads in a tube then measured out to approximately 3mg of powered tissue into tin capsules and sent off to the University of New Mexico Center for Stable Isotopes for analysis.

References

1. Forrestel EJ, Donoghue MJ, Smith MD. Functional differences between dominant grasses drive divergent responses to large herbivore loss in mesic savanna grasslands of North America and South Africa. Journal of Ecology. 2015 May;103(3):714-24.
2. Forrestel EJ, Donoghue MJ, Smith MD. Convergent phylogenetic and functional responses to altered fire regimes in mesic savanna grasslands of North America and South Africa. New Phytologist. 2014 Aug;203(3):1000-11.
