# Supplementary material for: Exploring the impact of trait number and type on functional diversity metrics in real-world ecosystems
Source: PLoS One. 2022 Aug 25;17(8):e0272791. doi: 10.1371/journal.pone.0272791 (PMC9409596; doi:10.1371/journal.pone.0272791)
Supplement: Attachment — Submitted filename: FDiv_response to reviewers.docx [file pone.0272791.s002.docx]

Dear Iván Prieto Aguilar, Ph.D.,

Re: Manuscript ID: PONE-D-21-22835

We thank you for your continued interest in our registered report and we are grateful for the opportunity to resubmit a revised manuscript. We thank you and the two reviewers for providing suggestions that will improve this manuscript. Both reviewers agreed that this study will be a valuable resource for ecological studies using trait-based functional diversity measures and that by increasing the scope of this study, we can make this contribution even more valuable.

In our revision, we addressed the key comments raised by the reviewers:

1) *Concern over using only the FD package, Gower dissimilarity, and functional richness limited the scope of methods to be tested.*

As Reviewer #1 points out, the FD package is still the most widely used way to calculate functional diversity in plant communities, but many methods for calculating functional diversity have recently emerged. Similarly, Gower dissimilarity is used when including categorical variables, but Euclidean dissimilarity is generally preferred when using only continuous variables. We have revised the manuscript to include tests of additional functional diversity metrics, such as FRich, FEve, FDisp, FDiv, Rao’s Q and hypervolumes using both Gower and Euclidean dissimilarity where data permits, alongside our initially proposed analyses.

2) *Methodological inconsistencies cause issues with both statistics and interpretation across sites.*

We agree with the reviewer’s concern over cross-site statistical models and the confounding issues that emerge when trying to compare 1x1m plots to 10x10m plots at Konza Prairie. To account for these issues, we will no longer include models for cross-site analyses and we are changing the source of plant community composition data at Konza Prairie to 1x1m plots to maintain consistency between sites. We believe this will facilitate more-accurate comparisons across the sites.

We also responded to the other comments from the reviewers in the text that follows..

We hope that this revised manuscript answers your concerns, and we are grateful for the helpful feedback. We think that the revised manuscript is a great improvement.

Thank you in advance for considering our resubmission.

Sincerely,

Timothy Ohlert and Kaitlin Kimmel

Reviewer #1: *The idea to analyse the existing methods using real- world data on different combinations of traits and/or scales of organisation sound interesting and currently demanding (i.e. Mammola et al. 2021). The main concern about this article is that the approach seems too simplistic for me both from theoretical and methodological approaches.*

*From a methodological perspective, it is true that FD package is one of the most famous tools to calculate FD, but in my opinion, it is due to the simplicity of the tool which makes that many researchers routinely use this method, without many theorical and/or mathematical consideration. The improve of the theoretical development of FD metrics has been accompanied by a proliferation of methods. In this regard, new tools have been developed to improve FD package. The FD package has many constraints that the authors overlook in the article and, from my point of view, should be taken into account or at least discussed, especially for a methodological article.*

>> We agree that the FD package has many issues and that many new packages improve upon the methods included in the FD package. Since the FD package is still the most commonly used method for calculating functional diversity, we will include other measures of functional diversity including FRic, FEve, FDisp, FDiv, Rao’s Q, and hypervolumes, and are happy to add others if the reviewers have specifics in mind. We have added this information to the text.

*For example, as the authors explain, the number of dimensions depends on the number of selected traits, however, If I´m not wrong, the FD package only uses two dimensions to calculate FD metrics, in this regard new tools have been developed to make a selection of the dimensions based on their redundancy (see de Bello et al. 2016; Maire et al. 2015; Gutiérrez-Cánovas et al. 2020).*

>> The reviewer is correct that the FD package uses two principal component axes for calculating its metrics. We believe that the number of traits used to calculate these principal component axes could influence the functional diversity indices. Therefore, we think it is still relevant to test these methods for the FD package along with other measures. We have added a more thorough explanation of the FD package and additional metrics in the text.

*In addition, functional richness often increases as new organisms are included in a group, if we compare Frich among studies, we are assuming the same sampling effort or the same sampling protocol, which I think is not the case in this article. Thus, to compare among studies a randomization could be desirable (Mammola et al. 2021).*

>> We agree that comparing among studies creates a conceptual issue with our data analyses. To avoid improper interpretation of our results, we will make separate models for each site instead of combining all sites into a single model. We have edited the text to reflect this change.

*Other constraint is the use of Gower distances with the FD package, as de Bello et al. 2020 explains “the Gower distance can however produce a multi-trait dissimilarity with a disproportional contribution of certain traits, particularly categorical traits and bundle of correlated traits reflecting similar ecological functions. Hence categorical traits will contribute more to the multi-trait dissimilarity”. In de Bellos´s article is well explained the constraints of use the Gower distance with FD package. In fact, I don´t really understand why the authors use Gower and not Euclidian distances because If I´m right there is only one categorical trait (growth form in Konza), why don´t remove it?*

>> Since categorical traits are some of the most popular and easily accessible traits (such as photosynthetic pathway included for both Sevilleta and Konza Prairie), it is important to test these methodological patterns in a way that incorporates categorical traits. This limits us to using Gower distance. That said, we agree that the Euclidean distance is also widely used and even preferred when only continuous traits are involved. Therefore, we will use both Gower and Euclidean distance in order to account for both the incorporation of categorical traits afforded by Gower distance and the favorability of Euclidean distance with continuous traits which will expand the usefulness of our findings. We have made this change in the text.

*From a theorical perspective, in the last five years a plethora of more sophisticated methods has been developed to represent the functional diversity, I don´t really know why to use FD package is the best option I really miss a strong dissertation about why the selected FD indices are the best choice. For instance, Frich are very sensitive to outliers, the space within extreme values of a convex hull is assumed to be homogeneous and also strongly depends of the number of species and traits, so why Frich should be a good index to compare Functional diversity among different studies or for meta-analyses? Why Functional divergence and not Rao or functional dispersion? In summary, the introduction needs more strong theoretical framework and less generalizations (such as Lines 59-60, 62-63, 74-75).*

>> A focus of our analysis is to highlight potential shortcomings in common functional diversity indices in order for researchers to account for these issues in their future use of these indices. Therefore, we think it is important to test the methodologies of these indices which may emphasize some of their weaknesses. To that end, we are also including additional metrics to test which we believe are likely to be better choices for quantifying functional diversity.

*References*

*de Bello, F., Botta‐Dukát, Z., Lepš, J., & Fibich, P. (2021). Towards a more balanced combination of multiple traits when computing functional differences between species. Methods in Ecology and Evolution, 12(3), 443-448.*

*de Bello, F., Carmona, C. P., Lepš, J., Szava‐Kovats, R., & Pärtel, M. (2016). Functional diversity through the mean trait dissimilarity: resolving shortcomings with existing paradigms and algorithms. Oecologia, 180, 933–940.* [*https://doi.org/10.1007/s00442*](https://doi.org/10.1007/s00442)*‐016‐3546‐0*

*Gutiérrez-Cánovas, C., Sánchez-Fernández, D., González-Moreno, P., Mateos-Naranjo, E., Castro-Díez, P., & Vilà, M. (2020). Combined effects of land-use intensification and plant invasion on native communities. Oecologia, 192(3), 823-836.*

*Maire, E., Grenouillet, G., Brosse, S., & Villeger, S. (2015) How many dimensions are needed to accurately assess functional diversity? A pragmatic approach for assessing the quality of functional spaces. Global Ecology and Biogeography, 24, 728-740.*

*Mammola, S., Carmona, C. P., Guillerme, T., & Cardoso, P. (2020). Concepts and applications in functional diversity. Functional Ecology.*

Reviewer #2: *General Comments*

*This is a Registered Report Protocol, which is a new publication type for me. The proposed work rests heavily on simulation-like trait calculations in three different sites to test how functional diversity metrics respond to  the number and correlation of traits used in different grassland ecosystems.*

*I have some fairly straightforward stats concerns listed in the methods comments below. Largely, I am concerned with treating the sites together in the statistical modeling, given different scales of vegetation monitoring; different surveyors estimating the ever subjective visual cover / abundance; and the different sets of traits. I think if each site was separated and analyzed only, more nuanced could be derived from each site result in how the trait selection and overall community structure may influence functional diversity metrics. The main question is about trends in diversity metrics based on trait number, and that could be answered while keeping separate models.*

>> We agree with this point, we had debated the best model structure and ended on the combined model because we thought it might be more generalized and prescriptive for further use of these methods at other sites. However, the reviewer raises many good points about splitting the models and we think that qualitative comparison of results from each site will yield similarly helpful information without the problems that cross-site models pose. We have made this change in the text

*In addition, this is a simulation-based study, and I feel that it is well-positioned to explore a wider set of questions given the rich data behind it. In particular, Lines 186 – 187 highlight an important component that would be fairly straightforward to code and include, and would dig concretely into some of the uncertainty around functional metrics. Without it, the study feels a little limited.*

>> The reviewer is correct to point out that intraspecific trait variation is an important dimension of trait diversity within communities. Failure to account for intraspecific trait variation is most problematic when trait values collected in one region of the world are ascribed to a different population of that species. Our data avoid this issue because the traits were collected at the site in which the plant community data were collected, substantially limiting intraspecific variation. Though testing the effects of intraspecific trait variation is outside the scope of our study due to limitations of the collected data, we have changed the text to emphasize its importance to the field.

*Introduction*

*Lines 30-31: I think lots of attention has been given to assumptions in this space, which you highlight in the next few sentences. I suggest removing this sentence or toning down the language.*

>> We have removed this sentence.

*Methods*

*The scale of monitoring differs between data sets, with Konza at 10m2 and the other two at 1m2. Does this likely impact diversity estimates and outcomes of this study? It would certainly impact species richness estimates. How have similar studies dealt with the issue (perhaps species diversity studies have dealt with this explicitly somehow)? Because you model all of the sites together in a single model, I find this concerning. Konza is uniquely large, so including site as a fixed effect does not cover scale of monitoring.*

>> We agree that comparing plots of different sizes obscures the effects of the study’s focus, number and correlation of traits used to calculate metrics. Therefore, we are changing our source for community composition data at the Konza Prairie site to be from an observational dataset with 1x1m plots. We have changed the text to reflect this.

*Given that you are looking for trends in the metrics within a site, perhaps splitting them into separate models, or standardizing the response values, may deal with this potential variation. Modeling them separately would also allow you to explore the different effect size of the trait number predictor between sites, the variation at different trait number levels within sites, and the role that your different sets of traits may play in influencing the trends.*

>> We agree, and we will split out models for each site individually instead of combining them. We have changed the text to reflect this.

*I also feel that it is hard to compare visual cover, as it is such a subjective metric and can vary so highly, especially between dry and mesic communities. The methods mention that in at least evenness, relative abundance is used. Can it also be used in the FDis calculations? If the models are split by site, this might not be an issue.*

>> We agree with this point and will use relative abundance for all abundance-weighted calculations. We had meant to include this detail in the methods and did not. Thank you for catching this.

*Lines 214 – 216: I’m not sure what “characterize trait type” means here.*

>> We have reworded this in the text for clarity.

*Lines 226 – 228: Models, ideally, should be defined a priori or at least based on clear ecological reasoning. Is there a particular ecological reason that you chose to test linear, log, and quadratic fits?*

>> Thank you for bringing this up. We agree that most models should be based on ecological reasoning, however, the metrics we are testing do not necessarily have to align with an ecological phenomenon. Because of this, we decided to test a range of model fits (linear, quadratic, cubic, and quartic) consistent with the methodology of Legras et al. 2020, and will allow us to detect non-linearities specifically. The shapes of these fits should not have ecological significance, per se, but instead methodological significance as to whether metrics saturate or become exponentially less similar based on the number and correlation of traits used. Since these metrics have not been tested with natural communities, we do not have expectations for the shapes of the fits except for those laid out in Legras et al. We have edited the text to reflect this change.

*Lines 228 – 229: I must have misread, but I thought each plot only had one value? (Lines 142; 153-155; 163).*

>> Though each plot only includes a single year of the community data, each plot will be used multiple times to generate the functional diversity indices using different numbers of traits. Therefore, we do not need to account for repeated measures in time but do need to account for repeated values of functional diversity calculated from the same plot.
